# Transcriptional Regulation of Carotenoid Biosynthesis in Plants: So Many Regulators, So Little Consensus

**DOI:** 10.3389/fpls.2019.01017

**Published:** 2019-08-09

**Authors:** Lauren Stanley, Yao-Wu Yuan

**Affiliations:** Department of Ecology and Evolutionary Biology, University of Connecticut, Storrs, CT, United States

**Keywords:** carotenoid biosynthesis, transcriptional regulation, photosynthetic tissue, flowers, fruits, seeds, roots

## Abstract

In plants, the carotenoid biosynthesis pathway (CBP) is essential for the production of photosynthetic and protective pigments, plant hormones, and visual/olfactory attractants for animal pollinators and seed dispersers. The regulation of carotenoid biosynthesis at the transcriptional level is vitally important for all of these functions and has been the subject of intensive research. Many putative transcriptional regulators, both direct and indirect, have been identified through conventional mutant analysis, transcriptome profiling, yeast one-hybrid screening, and candidate gene approaches. Despite this progress, our understanding of the transcriptional regulation of carotenoid biosynthesis remains fragmented and incomplete. Frequently, a stimulus or regulator is known, but the mechanism by which it affects transcription has not been elucidated. In other cases, mechanisms have been proposed (such as direct binding of a CBP gene promoter by a transcription factor), but function was tested only *in vitro* or in heterologous systems, making it unclear whether these proteins actually play a role in carotenoid regulation in their endogenous environments. Even in cases where the mechanism is relatively well understood, regulators are often studied in isolation, either in a single plant species or outside the context of other known regulators. This presents a conundrum: why so many candidate regulators but so little consensus? Here we summarize current knowledge on transcriptional regulation of the CBP, lay out the challenges contributing to this conundrum, identify remaining knowledge gaps, and suggest future research directions to address these challenges and knowledge gaps.

## Introduction

Carotenoids are red, orange, and yellow pigments produced by photoautotrophic organisms. In the green tissues of plants, carotenoids are essential for light capture, photoprotection, and stabilization of the photosynthetic apparatus (Frank and Cogdell, 1996; Hashimoto et al., 2016). Leaf carotenoids are therefore synthesized in tight coordination with chlorophylls, and their composition is remarkably conserved across higher plants (Goodwin and Britton, 1988; Meier et al., 2011). In addition to their integral roles in photosynthesis, carotenoids accumulate as secondary metabolites in many flowers and fruits to attract pollinators and seed dispersers. Due to their dispensable nature in non-green tissues, these pigments often differ drastically in composition and concentration between species or even between varieties of the same species (Moehs et al., 2001; Bradshaw and Schemske, 2003; Nielsen et al., 2003; Giovannoni, 2007; Ha et al., 2007; Chiou et al., 2010; Yamagishi et al., 2010; Yamamizo et al., 2010; Zhang et al., 2015). Floral and fruit carotenoids can also be cleaved to produce volatile compounds (i.e., scents and flavors), which further enhance plant–animal interactions (Dudareva et al., 2006). Finally, carotenoids serve as precursors for the synthesis of the plant hormones abscisic acid (ABA) and strigolactones, as well as other apocarotenoids that are involved in many developmental processes and stress responses (Cutler et al., 2010; Hou et al., 2016; Jia et al., 2017).

Because of their critical importance in the physiology, development, ecology, and evolution of plants, carotenoid metabolism and function have been intensively studied. The highly conserved carotenoid biosynthesis pathway (CBP) has been characterized in many plants (reviewed in Ruiz-Sola and Rodríguez-Concepción, 2012). In recent years, attention has turned to the regulation of carotenoid accumulation at multiple levels: transcriptional, post-transcriptional, post-translational, storage/degradation, and feedback regulation by end products. This has led to the discovery of numerous carotenoid regulatory mechanisms such as the post-translational regulation of phytoene synthase (PSY) by Orange (Or) (Lu et al., 2006; Zhou et al., 2015), the catabolism of carotenoids by carotenoid cleavage dioxygenases (CCDs) and 9-cis-epoxycarotenoid dioxygenases (NCEDs) (e.g., Auldridge et al., 2006; Ohmiya et al., 2006; Vallabhaneni et al., 2010), and feedback regulation by apocarotenoid-derived signaling molecules (e.g., Avendaño-Vásquez et al., 2014).

**Figure 1:**
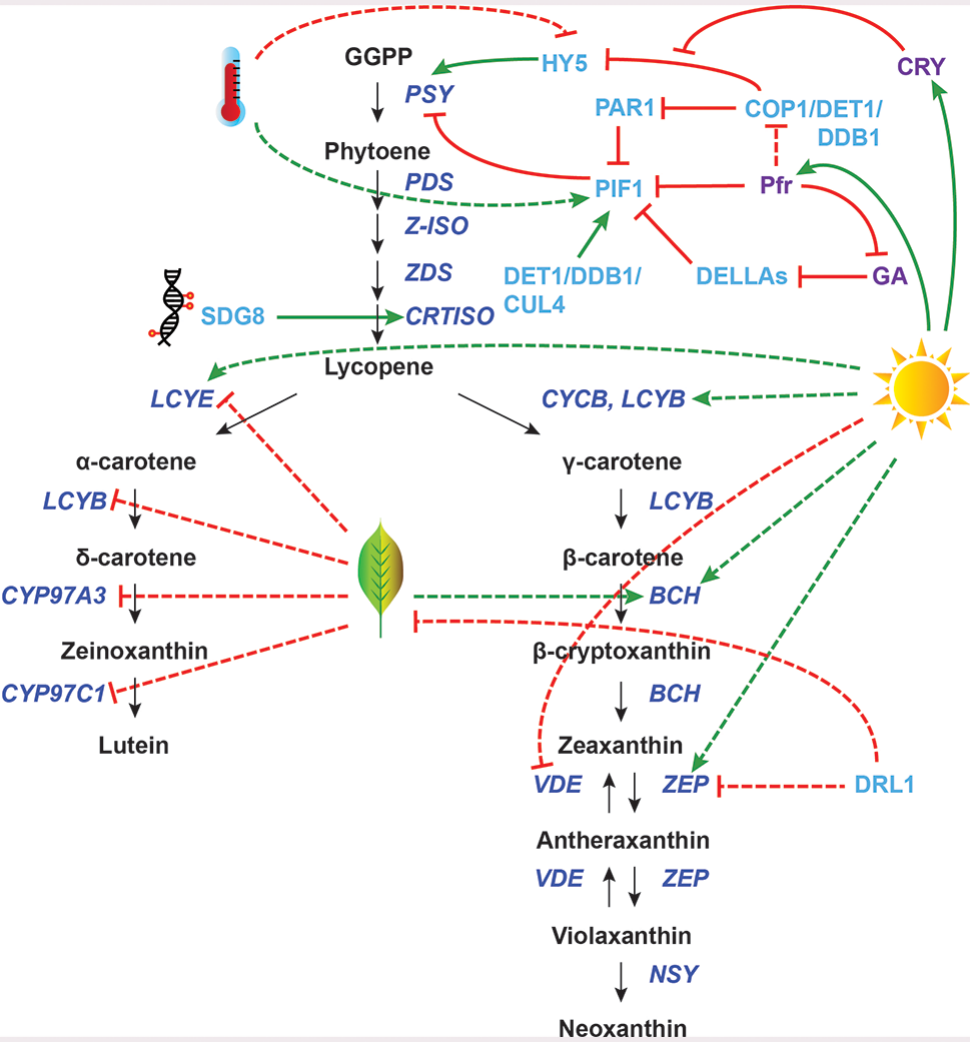
Transcriptional regulation of carotenoid biosynthesis pathway (CBP) genes in photosynthetic tissues. The regulation of CBP genes in response to light (sun), senescence (leaf), and high temperature (thermometer) and by epigenetic controls (DNA) is shown. The carotenoid biosynthesis pathway is in black, with carotenoid biosynthesis genes indicated in dark blue. Carotenoid regulators discussed in the paper are shown in light blue, with other regulators in purple. Green arrows indicate positive regulation, while blunt red arrows indicate negative regulation. Solid lines show direct interactions, while dotted lines show indirect/unknown interactions. GGPP, geranylgeranyl pyrophosphate; *PSY*, phytoene synthase; *PDS*, phytoene desaturase; *Z-ISO*, 15-cis-zeta-carotene isomerase; *ZDS*, zeta-carotene desaturase; *CRTISO*, carotene isomerase; *LCYB*, lycopene beta-cyclase; *CYCB*, chromoplast-specific lycopene beta-cyclase; *BCH*, beta-carotene hydroxylase; *ZEP*, zeaxanthin epoxidase; *VDE*, violaxanthin deepoxidase; *NSY*, neoxanthin synthase; *LCYE*, lycopene epsilon-cyclase; *CYP97A3*, cytochrome P450–type beta-hydroxylase; *CYP97C1*, cytochrome P450–type epsilon-hydroxylase.

In this review, we will focus on the transcriptional regulation of carotenoid biosynthesis genes. For other aspects of carotenoid regulation, we refer readers to several recent reviews (Cazzonelli and Pogson, 2010; Yuan et al., 2015; Nisar et al., 2015; Liu et al., 2015a; Hou et al., 2016; Li et al., 2016; Enfissi et al., 2017; Llorente et al., 2017; Sun et al., 2018a; Ohmiya et al., 2019). In this paper, “transcriptional regulation” of carotenoid biosynthesis genes simply refers to altered transcript abundance in response to a stimulus or as a result of the mutation, knockdown, or overexpression of another gene (e.g., transcription factor, chromatin remodeler). Additionally, we use the term “CBP genes” to refer to the core CBP, from *PSY* to *NSY* (see **Figure 1** for a schematic of the CBP). Upstream non-carotenoid specific genes [mevalonate (MVA) or methylerythritol phosphate (MEP) pathways], genes of the side branches leading to the production of hormones and apocarotenoids, and genes necessary for the production of uncommon carotenoids (e.g., capsanthin, capsorubin, astaxanthin), are not discussed in detail.

We have organized this review by tissue type because carotenoids serve unique functions in photosynthetic tissues, fruits, flowers, seeds, and roots and because the literature is already somewhat structured in this manner. For example, tomatoes are considered the model system for carotenoid biosynthesis in fruits, and *Arabidopsis* for that in leaves. Even in narrowing the scope to just transcriptional regulation, this review covers about 40 putative regulators of carotenoid biosynthesis genes (**Table S1**). However, there is little overlap of these numerous regulators between studies of different tissue types or different plant species. In other words, very few of these putative regulators seem to have a conserved function in the transcriptional control of carotenoid biosynthesis genes across tissue types or plant species. We lay out some of the challenges contributing to this conundrum, identify remaining knowledge gaps, and suggest research directions to address these challenges and knowledge gaps in the coming years.

### Photosynthetic Tissues

Carotenoids are an integral part of the light harvesting apparatus, capturing light energy and protecting the photosynthetic apparatus from damaging reactive oxygen species (ROS) formed during photosynthesis (Demmig-Adams and Adams, 1996; Pogson et al., 1998; Niyogi, 1999; Baroli and Niyogi, 2000; Dall’Osto et al., 2007). These pigments may also play an important role in temperature stress by scavenging ROS produced by photosystem II (PSII) in extreme temperatures and stabilizing thylakoid membranes (Havaux, 1998; Pospíšil, 2016). Additionally, the developmental program for leaf senescence requires carotenoid precursors for the production of ABA and strigolactones (Nambara and Marion-Poll, 2005; Snowden et al., 2005; López‐Ráez et al., 2008; Alder et al., 2012; Ueda and Kusaba, 2015). Transcriptional regulation of the CBP genes in photosynthetic tissues is therefore highly influenced by light, temperature, and developmental cues.

#### Light

The light signaling machinery of plants has been extensively characterized in *Arabidopsis* (e.g., Delker et al., 2014; Dong et al., 2014; reviewed in Jiao et al., 2007; Lau and Deng, 2012), and many key regulatory genes have been identified. One such gene, *Phytochrome Interacting Factor 1* (*PIF1*), is perhaps the best-understood transcriptional regulator of carotenoid biosynthesis. During seedling deetiolation, phytochromes are activated by red light and move from the cytoplasm into the nucleus to interact with signaling components. The bHLH transcription factor PIF1, which represses *AtPSY* expression in the dark, is phosphorylated by phytochromes upon photoactivation and subsequently degraded by the proteasome (Bae and Choi, 2008; Shen et al., 2008; Shin et al., 2009). This initiates the rapid de-repression of *AtPSY* as well as genes involved in chlorophyll biosynthesis and chloroplast development.

Toledo-Ortiz et al. (2010) showed that *Arabidopsis* PIF1 binds directly to a G-box element in the *AtPSY* promoter in both *in vitro* and *in vivo* assays and demonstrated that this binding leads to transcriptional repression. PIFs also contribute to the regulation of *AtPSY* in mature plants during their daily light/dark cycles. In fully deetiolated plants grown under short-day conditions, higher levels of carotenoids and *AtPSY* transcripts were found in *pif* mutants than in wild-type plants (Toledo-Ortiz et al., 2010).

Another important player in light signaling is the bZIP transcription factor Long Hypocotyl 5 (HY5), which acts antagonistically to PIF1 during photomorphogenesis. HY5 activates carotenoid and chlorophyll biosynthesis genes, as well as genes involved in chloroplast development and cotyledon expansion. Unlike PIF1, which is stabilized in the dark by the DET1/DDB1/CUL4 complex, HY5 is stabilized by light (the COP1/DDB1/CUL4 complex targets HY5 for degradation in the dark) (Shi et al., 2015; Zhu et al., 2015). Interestingly, HY5 and PIF1 bind to the same G-box element of the *AtPSY* promoter, which serves as a relatively simple switch to promote deetiolation upon illumination. This switch also functions in the daily light/dark cycles of mature plants (Toledo-Ortiz et al., 2014).

PIFs are also involved in shade-triggered reduction of carotenoid accumulation in *Arabidopsis* leaves, through an HY5-independent mechanism. When there is a low red/far red (R/FR) ratio of light in shade conditions, Phytochrome Rapidly Regulated 1 (PAR1), a bHLH co-factor, is upregulated and induces *AtPSY* expression by physically interacting with PIF1 and preventing it from sitting on the *AtPSY* promoter (Bou-Torrent et al., 2015).

Carotenoid biosynthesis is also induced when greening is de-repressed in the dark, which can be achieved through the blockage of gibberellic acid (GA) biosynthesis (Rodríguez-Villalón et al., 2009; Toledo-Ortiz et al., 2010). GA negatively regulates DELLA proteins, which in turn negatively regulate PIFs. In *Arabidopsis* GA biosynthesis mutants, *AtPSY* transcript levels in etiolated seedlings are elevated relative to the wild type. In “double” mutants lacking both GA and DELLAs, this response is repressed. Treatment of wild-type plants with a GA inhibitor reduced PIF1 binding to the G-box in the *AtPSY* promoter (Cheminant et al., 2011).

While the PIF1/HY5 regulatory mechanism is relatively well understood, there is still much to be learned about the transcriptional regulation of carotenoid biosynthesis during deetiolation. For instance, many other carotenoid biosynthesis genes are de-repressed during photomorphogenesis in *Arabidopsis*, such as *AtBCH2*, *AtZEP*, and *AtLCYE* (which are constitutively de-repressed in *pif* mutants). Of these, only *AtBCH2* has a G-box in its promoter, but this G-box is not bound by PIF1 (Toledo-Ortiz et al., 2010). Additionally, truncated *AtPSY* genes lacking G-boxes in Arabidopsis are still light responsive (Welsch et al., 2003), indicating that there are other factors involved in light responsiveness unrelated to the PIF pathway and/or that PIFs may bind other elements.

Indeed, a chromatin immunoprecipitation–microarray (ChIP–chip) analysis in *Arabidopsis* seeds showed that PIF1 binds to 748 sites, only 59% of which contain G-box elements (Oh et al., 2009); additionally, only a small fraction of G-box elements in the *Arabidopsis* genome have been shown to be bound by PIFs (Kim et al., 2016). PIF1 has been shown to bind PIF binding E-box (PBE) elements *in vitro*, though this interaction is relatively weak (Kim et al., 2008; Pfeiffer et al., 2014). PIF1-interacting transcription factors may facilitate the targeting of PIF1 to specific sites, particularly those containing multiple G-boxes and/or G-box coupling elements (GCEs) (Kim et al., 2016). Thus, non-canonical PIF binding sites may play a role in PIF1 regulation of other CBP genes.

Another thing to consider is that PIF1 is certainly not a specific carotenoid regulator: it has been shown to directly regulate the chlorophyll biosynthesis gene *AtPOR* by binding its promoter and to indirectly regulate other genes in that pathway (Moon et al., 2008). This may account in part for the tight coordination between chlorophyll and carotenoid biosynthesis in green tissues during photomorphogenesis. However, for PIF1 to function in chromoplast-containing tissues, the regulation of carotenoid and chlorophyll biosynthesis must be decoupled (see the "Fruits" section).

The intensity of light affects both carotenoid concentration and composition (Hirschberg, 2001). High light stress produces ROS such as triplet chlorophyll and singlet oxygen, which can be deactivated by carotenoids. Additionally, excess excitation energy in the photosystems can be effectively dissipated by carotenoids, particularly zeaxanthin (Dall’Osto et al., 2012; Jahns and Holzwarth, 2012). High light causes a rapid decrease in lumen pH, which increases violaxanthin deepoxidase (VDE) enzyme activity, converting violaxanthin to zeaxanthin (**Figure 1**). Although this interconversion between violaxanthin and zeaxanthin (i.e., the xanthophyll cycle) is regulated post-translationally by activation and inactivation of the VDE enzyme (Müller et al., 2001), high light does induce transcriptional changes of CBP genes as well. For example, the ratio of *LCYB* to *LCYE* transcripts increases fivefold in both *Arabidopsis* and tomato leaves in high light relative to low light (Hirschberg, 2001), which channels metabolic flux through the branch of the CBP that produces zeaxanthin (**Figure 1**). *AtBCH2* transcription is also upregulated by high light treatment in *Arabidopsis* (Rossel et al., 2002), likely enhancing the metabolic flow towards xanthophylls as well. However, the transcription factors responsible for these CBP gene expression changes remain unknown.

Ultraviolet B (UV-B) light also triggers the production of carotenoids, which are directly linked to photoprotection of the photosynthetic apparatus (Middleton and Teramura, 1993). Irradiation of *Arabidopsis* plants with UV-B causes slight decreases in lutein and β-carotene content but a substantial increase in zeaxanthin. Correspondingly, the expression of *AtPSY*, *AtZDS*, and *AtBCH1/2* is enhanced. Loss-of-function *AtLYCE* (*LUT2*) mutants accumulate more β-carotene branch xanthophylls compared to the wild type and consequently show decreased DNA and oxidative damage under UV-B light (Emiliani et al., 2018). Interestingly, the UV-B response pathway and the photomorphogenesis pathway share several common components. For example, the active form of UV RESPONSE LOCUS 8 (UVR8), a UV-B–specific photoreceptor, directly interacts with COP1 and regulates *HY5* expression (Brown et al., 2005; Brown and Jenkins, 2008; Cloix and Jenkins, 2008; Favory et al., 2009; Rizzini et al., 2011). Therefore, it would not be surprising if the transcriptional regulators of the CBP genes downstream of *AtPSY* (e.g., *AtZDS*, *AtBCH1/2*) turned out to be the same for both UV-B response and photomorphogenesis.

#### Temperature

The PIF1/HY5 switch can also control *AtPSY* expression in response to temperature cues (Toledo-Ortiz et al., 2014). In addition to being stabilized by light, HY5 is stabilized by cold temperatures (Catalá et al., 2011). In a ChIP assay, both the *AtPSY* and *AtVDE* promoters were preferentially bound by HY5 at low compared to ambient temperatures. Furthermore, the rapid increase of HY5 and decrease of PIF1 protein levels when etiolated *Arabidopsis* seedlings were exposed to light was more robust at lower temperature (Toledo-Ortiz et al., 2014). This would lead to higher expression of *AtPSY* at lower temperatures. These findings are echoed by experiments in maize (*Zea mays*), which showed that *ZmPSY1* and *ZmPSY2* expression decreases at high temperatures in both light and dark conditions (Li et al., 2008a).

The sensitivity of *PSY* transcript levels to temperature cues indicates that transcriptional regulation of the CBP may be partially responsible for temperature stress responses. It makes sense that high light and low temperature responses would overlap, because the consequences of these stressors are similar: they both produce ROS and prevent the repair of PSII damage (reviewed in Szymańska et al., 2017). Thus, PIF1/HY5 regulation of *PSY* may be an important mechanism for increasing carotenoids to scavenge ROS. However, high temperature stress, which also produces damaging ROS, reduces the expression of *PSY*, indicating that transcriptional regulation of this gene is not responsible for high temperature stress response.

#### Senescence

Leaf senescence is a developmentally controlled process leading eventually to organ death. The breakdown and recycling of macromolecules from senescing leaves allow plants to reallocate resources to reproduction or new growth (Gan and Amasino, 1997; Lim et al., 2007). One of the most prominent phenotypes during senescence is leaf yellowing due to the breakdown of pigments in chloroplasts (Ougham et al., 2005). Although all photosynthetic pigments are eventually broken down, chlorophylls are usually lost more rapidly than carotenoids. There are also changes in the composition of carotenoids during senescence: while all decline, lutein remains at relatively stable levels compared to neoxanthin, violaxanthin, and antheraxanthin (Biswal, 1995; Britton and Young, 1989). This is perhaps due to the cleavage of β-carotene branch carotenoids for the production of strigolactones and ABA, which further promote leaf senescence (Yang et al., 2003; Ueda and Kusaba, 2015).

The transcription of CBP genes changes dramatically during leaf senescence. In an *Arabidopsis* microarray analysis of senescing leaves, *AtLCYE*, *AtCYP97C1*, and *AtCYP97A3* expression drops, reducing flux through the α-carotene branch of the pathway. This is followed by the induction of *AtBCH1*, which might be important for downstream hormone production (Breeze et al., 2011). Similar trends can also be seen in woody perennial plants: in aspen trees (*Populus tremula*), *PtBCH2* is significantly induced by autumn senescence (Andersson et al., 2004).

The only known potential regulator of CBP genes during leaf senescence is *DRL1* from grapevine (*Vitis vinifera*), encoding a NAC transcription factor. Overexpression of *DRL1* in tobacco has been shown to delay leaf senescence and decrease ABA levels. The expression of *NtZEP1* and carotenoid cleavage genes is reduced in these transgenic plants (Zhu et al., 2019). However, the endogenous function of *DRL1* in grapevine is yet to be reported.

#### Other CBP Regulators in Photosynthetic Tissues

Besides *ZEP*, two other CBP genes downstream of *PSY* have potential known regulators in photosynthetic tissues. In *Arabidopsis* leaves, the Ethylene Response Factor (ERF) transcription factor RELATED TO AP2 2 (RAP2.2) was shown to bind the *AtPSY* and *AtPDS* promoters *in vitro* (Welsch et al., 2007). However, overexpression of *AtRAP2.2* in *Arabidopsis* leaves did not result in higher *AtPSY* or *AtPDS* messenger RNA (mRNA) levels, nor a change in carotenoid concentration. A knockout mutant of *AtRAP2.2* was not available, probably due to lethality. These results leave the endogenous function of *AtRAP2.2* in carotenoid regulation ambiguous.

*AtCRTISO* is another CBP gene in photosynthetic tissues with an identified regulator: the histone methyltransferase Set Domain Group 8 (SDG8). *sdg8* mutants produce low levels of *CRTISO* mRNA, which correlates with reduced trimethyl-H3K4 and increased dimethyl-H3K4 around the *CRTISO* transcription start site (Cazzonelli et al., 2009). Although this mechanism is well understood, SDG8 is certainly not a specific carotenoid regulator: mutation in this gene downregulates 85 other genes and causes broad pleiotropic effects, including increased shoot branching, reduced fertility, and early flowering. It is currently unknown whether the function of SDG8 in carotenoid biosynthesis is conserved across species.

As described above, only a few regulators of carotenoid biosynthesis in green tissues, such as PIF1 and HY5, have been well characterized and shown to directly regulate *PSY* in *Arabidopsis*. Additionally, their importance as regulators of photomorphogenesis, responses to daily light/dark cycles, and temperature has been established. However, we still know very little about what regulates most CBP genes downstream of *PSY* in green tissues.

### Fruits

The ripening developmental program of fleshy fruits involves changes in texture (alteration of cell wall composition, reduction in turgor pressure), flavor and aroma (alteration of volatiles, sugar/starch, and acid metabolism), and color (alteration of chlorophyll, carotenoid, and flavonoid content) (Klee and Giovannoni, 2011). Many economically important fruits (e.g., tomato, orange, papaya) produce copious carotenoids during ripening, and therefore, the transcriptional control of CBP genes during fruit ripening has attracted considerable research efforts.

#### Tomatoes

**Figure 2:**
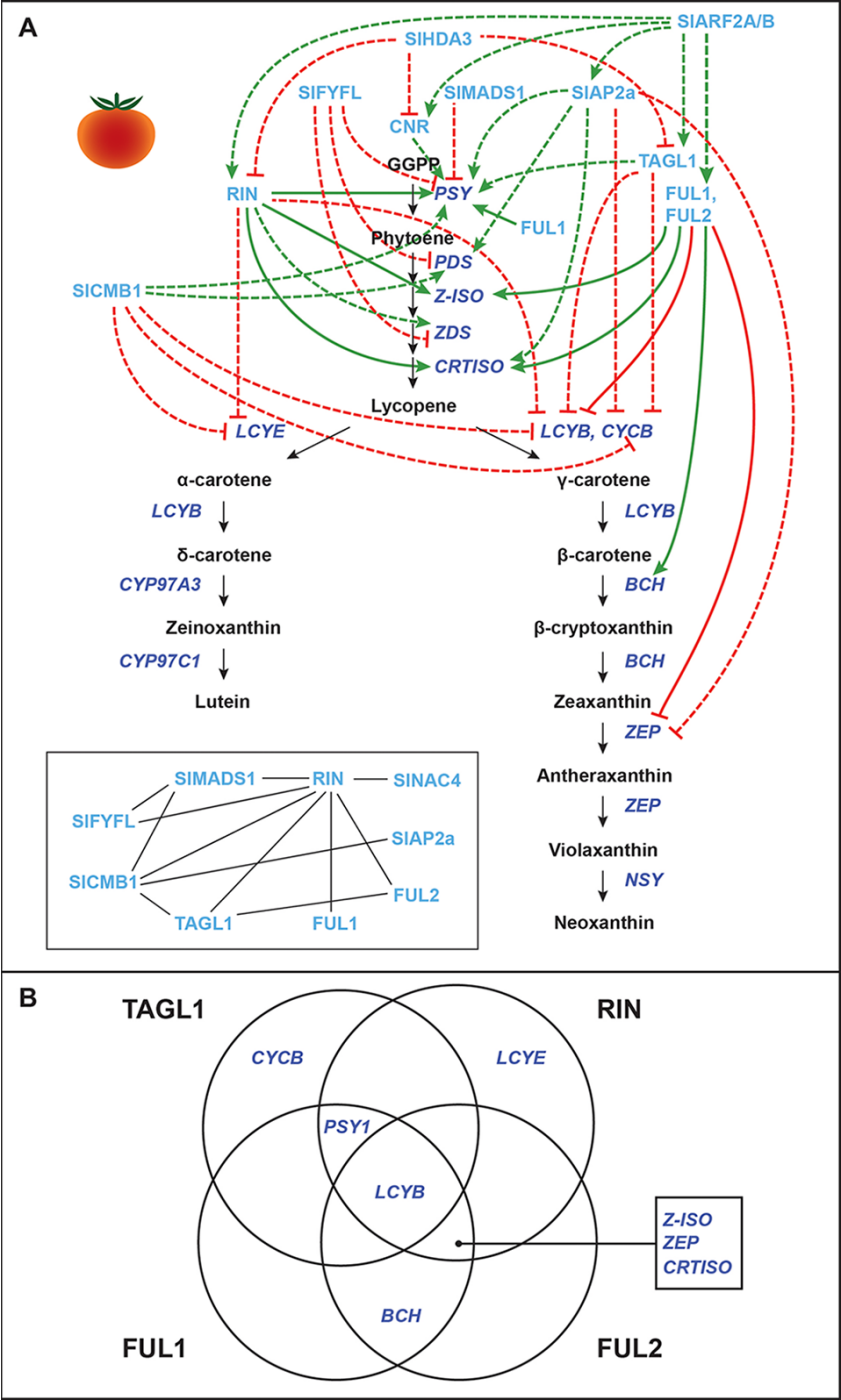
Transcriptional regulation of CBP genes in tomato fruits: “ripening quartet”–related proteins. **(A)** The regulation of CBP genes in tomato fruits. The carotenoid biosynthesis pathway is in black, with carotenoid biosynthesis genes indicated in dark blue. Carotenoid regulators discussed in the paper are shown in light blue. Green arrows indicate positive regulation, while blunt red arrows indicate negative regulation. Solid lines show direct interactions, while dotted lines show indirect/unknown interactions. Inset: protein–protein interactions between “ripening quartet” regulators. **(B)** Venn diagram showing the direct binding of TAGL1, RIN, FUL1, and FUL2 to CBP gene promoters.

**Figure 3:**
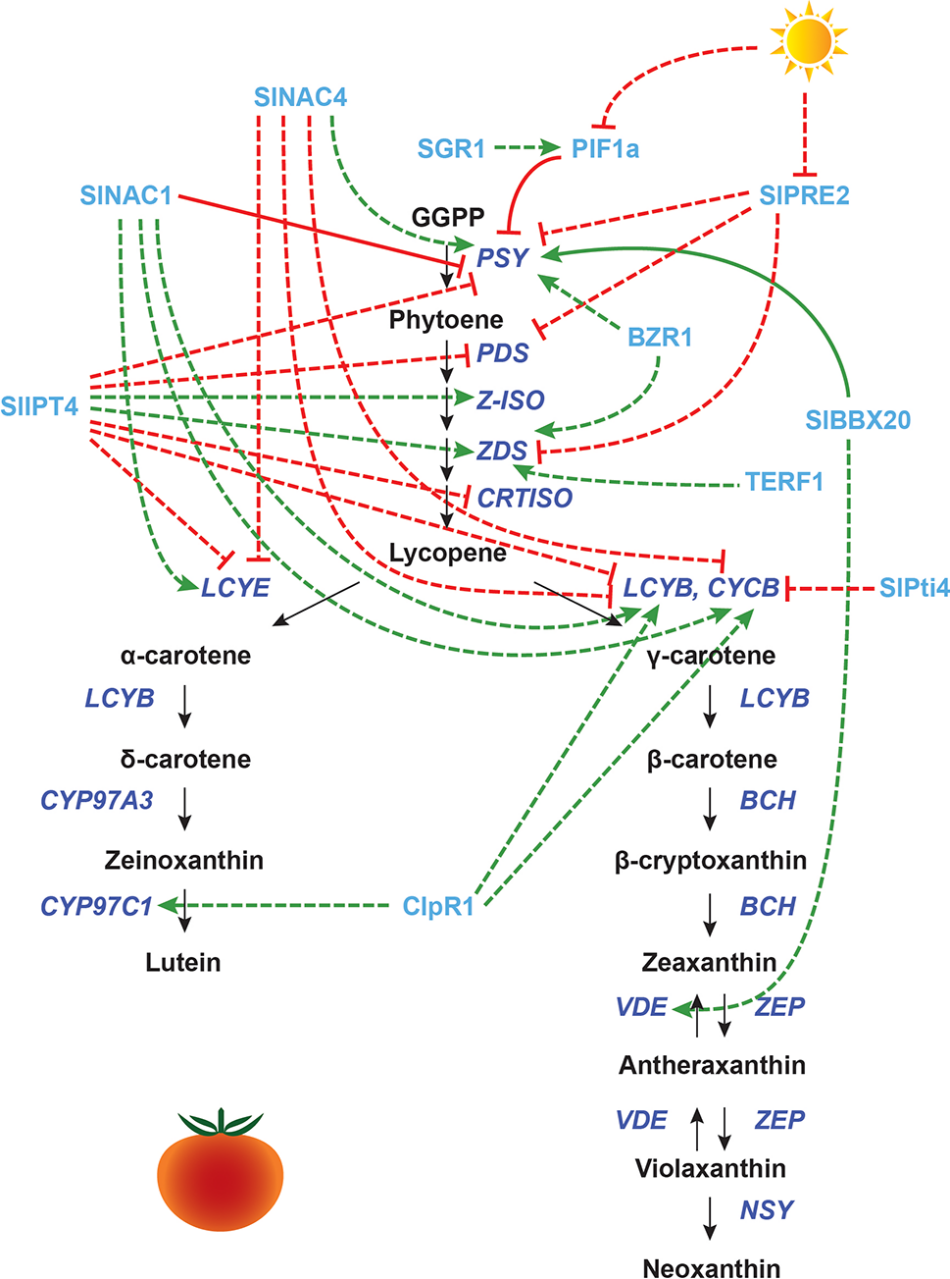
Transcriptional regulation of CBP genes in tomato fruits: other proteins. The regulation of CBP genes in tomato fruits. The carotenoid biosynthesis pathway is shown in black, with carotenoid biosynthesis genes indicated in dark blue. Carotenoid regulators discussed in the paper are shown in light blue. Green arrows indicate positive regulation, while blunt red arrows indicate negative regulation. Solid lines show direct interactions, while dotted lines show indirect interactions.

The foremost model for carotenoid regulation during fruit ripening is tomato (*Solanum lycopersicum*) (**Figures 2**–**3**), which primarily accumulates lycopene in mature fruits. During tomato fruit development, transcription of the early CBP genes *SlPSY1* and *SlPDS* increases, while the transcription of *SlLCYE* and *SlLCYB*, which convert lycopene to other downstream products, decreases (Pecker et al., 1992; Giuliano et al., 1993; Fraser et al., 1994; Corona et al., 1996; Ronen et al., 1999; Alba et al., 2005). Tomato fruits are climacteric, and thus, ethylene biosynthesis and signaling are necessary for the onset and completion of ripening (reviewed in Liu et al., 2015b). CBP gene regulation is also tightly coupled with these processes, and many of the regulators that affect CBP gene expression also affect other aspects of ripening. These may therefore be considered general regulators of ripening, oftentimes functioning far upstream of the CBP genes.

Several MADS-box ripening regulators affect the expression of CBP genes in tomatoes, and a ripening model similar to the floral quartet model has been proposed (**Figure 2**). In this model, different combinations of MADS-box proteins bind different target genes (Bemer et al., 2012; Shima et al., 2013; Fujisawa et al., 2014). These “ripening quartet” regulators include Tomato AGAMOUS-LIKE1 (TAGL1), Ripening Inhibitor (RIN), FRUITFULL1 (FUL1), and FUL2. These transcription factors have both overlapping and individual (but never antagonistic) contributions to the expression of CBP genes, with the total effect being the positive regulation of *SlPSY1*, *SlPSY2*, *SlZDS*, *SlZ-ISO*, *SlCRTISO*, and *SlBCH*, and the negative regulation of *SlLCYB*, *SlLCYE*, and *SlCYCB* (the chromoplast-specific paralogue of *SlLCYB*) (Vrebalov et al., 2002; Ito et al., 2008; Itkin et al., 2009; Vrebalov et al., 2009; Giménez et al., 2010; Fujisawa et al., 2011; Fujisawa et al., 2012; Fujisawa et al., 2013; Fujisawa et al., 2014; Martel et al., 2011; Bemer et al., 2012; Qin et al., 2012; Shima et al., 2013; Zhong et al., 2013). For the effects of each individual regulator, see **Figure 2A**.

These MADS-box proteins exert their effects over CBP gene transcription both directly by binding the promoters of some genes and indirectly by unknown mechanisms. Various studies have shown the promoter of *SlLCYB* to be bound by all four regulators; the promoter of *SlPSY1* to be bound by TAGL1, RIN, and FUL1; the promoters of *SlZ-ISO*, *SlCRTISO*, and *SlZEP* by RIN, FUL1, and FUL2; the promoter of *SlBCH* by FUL1 and FUL2; the promoter of *SlCYCB* by TAGL1; and the promoter of *SlLCYE* by RIN (Ito et al., 2008; Itkin et al., 2009; Vrebalov et al., 2009; Giménez et al., 2010; Fujisawa et al., 2011; Fujisawa et al., 2012; Fujisawa et al., 2013; Fujisawa et al., 2014; Martel et al., 2011; Bemer et al., 2012; Qin et al., 2012; Shima et al., 2013; Zhong et al., 2013; see **Figure 2B** for a graphical summary).

It should be mentioned that ChIP studies assessing RIN binding to target gene promoters have produced inconsistent results. Some studies have shown that the *SlPSY1* promoter is bound by RIN (Martel et al., 2011; Zhong et al., 2013; Fujisawa et al., 2013), while others have not detected this interaction or had inconclusive results (Fujisawa et al., 2011; Fujisawa et al., 2012; Fujisawa et al., 2013). Also, though FUL1 has been shown to bind the promoter of *SlPSY1* and promote its expression (Shima et al., 2013; Fujisawa et al., 2014), one study in which *FUL1* and *FUL2* were silenced showed that fruits did not have altered expression of *SlPSY1* (Bemer et al., 2012). The loss of FUL1 function may have been compensated by RIN and TAGL1, and thus, its endogenous role in regulating carotenoid biosynthesis remains unclear.

Other CBP-regulating MADS-box genes in tomato that interact with or regulate the ripening quartet are *SlMADS1*, *SlFYFL*, and *SlCMB1* (**Figure 2**). SlMADS1 and SlFYFL are both negative regulators of carotenoid biosynthesis, suppressing *SlPSY1* expression (SlFYFL also suppresses *SlPDS* and *SlZDS*) (Dong et al., 2013; Xie et al., 2014). SlCMB1 promotes the expression of *SlPSY1* and *SlPDS*, while suppressing *SlCYCB*, *SlLCYB*, and *SlLCYE* transcription (Zhang et al., 2018a).

Many other regulators play a role in CBP gene regulation during fruit ripening, especially those involved in hormone synthesis and signaling. SlAP2a (an APETALA2/ERF protein) positively regulates fruit ripening, promoting the expression of *SlPSY1*, *SlCRTISO*, *SlBCH*, and *SlPDS1*, and repressing *SlZEP1* and *SlCYCB* (Chung et al., 2010; Karlova et al., 2011). The overexpression of *Tomato Ethylene Response Factor 1 (TERF1)* induces *SlZDS* expression, perhaps *via* plastid-to-nucleus retrograde signaling (Wu et al., 2019). Silencing *LeHB-1* (encoding an HD-Zip transcription factor) inhibits fruit ripening and lycopene production, most likely through inhibition of ethylene biosynthesis (Lin et al., 2008). However, the transcript levels of CBP genes were not assessed in the *LeHB-1* silenced lines.

NAC family transcription factors involved in ethylene biosynthesis also affect the transcription of CBP genes: SlNAC4 positively regulates *SlPSY1* and negatively regulates *SlCYCB*, *SlLCYB*, and *SlLCYE* (Zhu et al., 2014), while SlNAC1 has the opposite effect (Ma et al., 2014; Meng et al., 2016). SlNAC1 has been shown by yeast one-hybrid assay to interact with the promoters of *SlPSY1* and ethylene biosynthesis genes (Ma et al., 2014).

Additionally, overexpression of *SlNAC1* increases the amount of ABA, potentially by providing the carotenoid precursors for its synthesis (Ma et al., 2014). In wild-type fruits, ABA production precedes ethylene production and may be an important trigger for ripening (Zhang et al., 2009). ABA application promotes several ripening processes, including carotenoid accumulation, by regulating transcription factors for ethylene biosynthesis and signaling (Mou et al., 2016). Another regulator related to ethylene–ABA is SlPti4 (also a member of the AP2/ERF superfamily). Silencing of *SlPti4* enhances ABA production while decreasing ethylene production, which induces the expression of *SlCYCB* and the consequent color change from red to orange (Sun et al., 2018b). Finally, the zinc finger transcription factor SlZFP2 inhibits fruit ripening by negatively regulating ABA biosynthesis (Weng et al., 2015). *SlZFP2*-overexpressing fruits accumulate more β-carotene and lycopene compared to the wild type, but it is unclear whether SlZFP2 actually regulates CBP gene expression, as the transcript levels of CBP genes were not reported in this study (Weng et al., 2015).

Besides ethylene and ABA, other plant hormones are also involved in tomato fruit ripening, with complex actions and interactions. Auxin acts antagonistically to ethylene, delaying ripening. Exogenous application of an auxin inhibitor to tomato fruits produces an effect similar to ethylene application, indicating that perhaps the presence vs. absence of auxin, not ethylene *per se*, determines ripening in tomato (Su et al., 2015). RNA interference (RNAi) mediated silencing of two paralogues of *Auxin Response Factor 2*, *SlARF2A* and *SlARF2B*, caused downregulation of *SlPSY1*, *SlPDS*, and *SlZDS* and upregulation of *SlLCYB1*, *SlLCYB2*, and *SlCYCB* (Hao et al., 2015). Several key ripening genes (e.g., *RIN*, *CNR*, *NOR*, *AP2a*, *TAGL1*, *FUL1/2*) were also downregulated in these RNAi lines, indicating that *SlARF2A/B* may regulate CBP gene expression through the ripening factors.

Brassinosteroid (BR) application to pericarp discs induces lycopene formation (Vardhini and Rao, 2002). Transgenic lines overexpressing *Brassinazole Resistant 1* (*BZR1*) have increased transcript levels of *SlPSY1* and *SlZDS* (Liu et al., 2014). Cytokinins (CKs) are also involved in ripening: SlIPT4, which catalyzes the rate-limiting step of CK biosynthesis, represses the expression of *SlPSY1* and *SlPDS*, while upregulating the expression of *SlZ-ISO* and *SlZDS* (Zhang et al., 2018b). Given that both BR and CK are isoprenoids synthesized using precursors from the MEP/MVA pathways, it is perhaps not too surprising that their biosynthesis and signaling affect carotenoid biosynthesis (Sakakibara, 2006; Andrade et al., 2017). Another hormone, jasmonic acid (JA), promotes lycopene accumulation and CBP gene expression, even in ethylene-insensitive mutants (Liu et al., 2012), but the mechanism is unknown.

Light is also an important regulator of CBP genes during tomato fruit development. Interestingly, the shading response seen in *Arabidopsis* leaves is utilized in tomatoes for fruit ripening. In an *in vivo* ChIP assay, SlPIF1a binds to a PBE-box located in the promoter of the tomato *SlPSY1* gene to repress its expression. This repression is only maintained when the R/FR light ratio is low. In an elegant experiment, Llorente et al. (2016) showed that the presence of chlorophyll in the immature green fruit pericarp acts as a self-shading mechanism, giving a low R/FR ratio. When developmentally triggered degradation of chlorophyll begins, the R/FR ratio increases, and *SlPSY* is de-repressed by phytochrome-mediated degradation of SlPIF1a.

Other light signaling components have been examined in tomato fruits. Mutations in the high pigment genes *HP1* (*DDB1*) and *HP2* (*DET1*) give increased amounts of chlorophyll in immature fruits and increased amounts of carotenoids in mature fruits (Mustilli et al., 1999; Levin et al., 2003; Lieberman et al., 2004). DDB1 and DET1, which are known to interact with PIF1/HY5 and regulate their protein levels in *Arabidopsis* leaves, do not appear to strongly affect the expression of CBP genes in tomato fruits. The high pigment levels are probably due to changes in plastid size and/or number that increase the storage capacity of carotenoids. CBP gene expression has been shown to be slightly altered in tomato *det1* mutants compared to wild type: in a transcriptome study, *SlPSY1*, *SlPDS*, and *SlLCYB* transcript levels were all slightly elevated in immature fruits, while *SlCYCB* transcript levels were reduced. At the mature red stage, *SlPSY1*, *SlZDS*, and *SlCYCB* were upregulated, but carotenoid biosynthesis is not at peak levels in mature fruit, and thus, these differences may not be developmentally relevant (Kolotilin et al., 2007).

Another light-responsive CBP regulator in tomato is *SlPRE2*, an atypical bHLH transcription factor whose expression is repressed in high light. When *SlPRE2* is overexpressed, it alters the growth of stems and leaves, promotes hypocotyl elongation, and downregulates chlorophyll biosynthesis genes as well as *SlPSY1*, *SlPDS*, and *SlZDS*. Transcript levels of *HY5* are also reduced, which could explain the low level of *SlPSY1* transcripts (Zhu et al., 2017a).

Other proteins appear to indirectly affect the transcription of CBP genes through plastid processes. Overexpression of the B-box protein BBX20 increases the chlorophyll and carotenoid content in tomato leaves and fruits, inducing *SlPSY1* and *SlVDE* expression. BBX20 was found to bind to a G-box in the *SlPSY1* promoter *in vitro* and interacts with DET1. Carotenoid content is probably enhanced because of both the increase in *SlPSY1* expression and an increased number of chloroplasts. *BBX20* overexpression does not affect carotenoid accumulation or CBP transcription in flowers, indicating that distinct mechanisms operate in fruits and flowers (Xiong et al., 2019).

The Clp protease *ClpR1* enhances transcript levels of *SlLCYB*, *SlCYCB*, and *SlCYP97C11*. This gene probably affects the transcription of carotenoid biosynthesis genes through its contributions to the chloroplast-to-chromoplast transition (D’Andrea et al., 2018). The Stay Green 1 (SGR1) protein represses *SlPSY1* expression (although the effect appears minor). When *SGR1* is knocked down, fruits have lower *PIF1* expression and altered ethylene signal transduction. SGR1 also interacts directly with SlPSY1 protein, and knockdown of this gene induces early chloroplast-to-chromoplast transition, indicating that this gene has many regulatory functions (Luo et al., 2013).

Epigenetic regulation is also crucial to fruit ripening and carotenoid biosynthesis in tomato. Zhong et al. (2013) showed that about 1% of the tomato genome shows differential methylation during fruit ripening by chemically inhibiting methyltransferases. For a review on epigenetic controls in tomato fruit ripening, see Giovannoni et al., 2017; for a review on epigenetic control of carotenoid biosynthesis, see Arango et al., 2016.

Colorless non-ripening (Cnr) tomato mutants do not express *SlPSY1* (Thompson et al., 1999, Eriksson et al., 2004) and thus do not produce lycopene. The CNR locus was shown to be a *SQUAMOSA Promoter Binding Protein–like (SPL)* gene, with the causal mutation occurring in the promoter. This mutation was an epimutation, with increased methylation in mutants (Manning et al., 2006). When wild-type tomato fruits are treated with a methylation inhibitor, they produce early-ripening red sectors (which have unmethylated CNR promoters). The sectors that remain green also remain hypermethylated, suggesting that methylation of ripening genes acts as a developmental block. *SlPSY1* transcripts were isolated from early-ripening sectors, suggesting that this fruit ripening mechanism is upstream of carotenogenesis and other ripening processes (Zhong et al., 2013). The CNR *SPL* gene might be a conserved carotenoid regulator across species. Constitutive expression of *AtmiR156b* (which silences *AtSPL3*, a CNR *SPL* homologue; Wang et al., 2009) produces excess amounts of lutein and β-carotene in *Brassica napus* seeds, though CBP gene expression was not assessed (Gandikota et al., 2007; Wei et al., 2010; Arango et al., 2016). None of the CBP genes has a sequence complementary to AtmiR156b, so this increase in carotenoids is indirect (Wei et al., 2010), likely through the *SPL* gene.

Another epigenetic regulator, the tomato histone deacetylase gene *SlHDA3*, negatively regulates *SlPSY1* expression, while positively regulating *SlCYCB*, *SlLCYB*, and *SlLCYE*. Ethylene biosynthesis genes and cell wall metabolism genes were also negatively regulated by *SlHDA3*, as were *RIN*, *Cnr*, and *TAGL1* (Guo et al., 2018).

#### Other Climacteric Fruits

**Figure 4:**
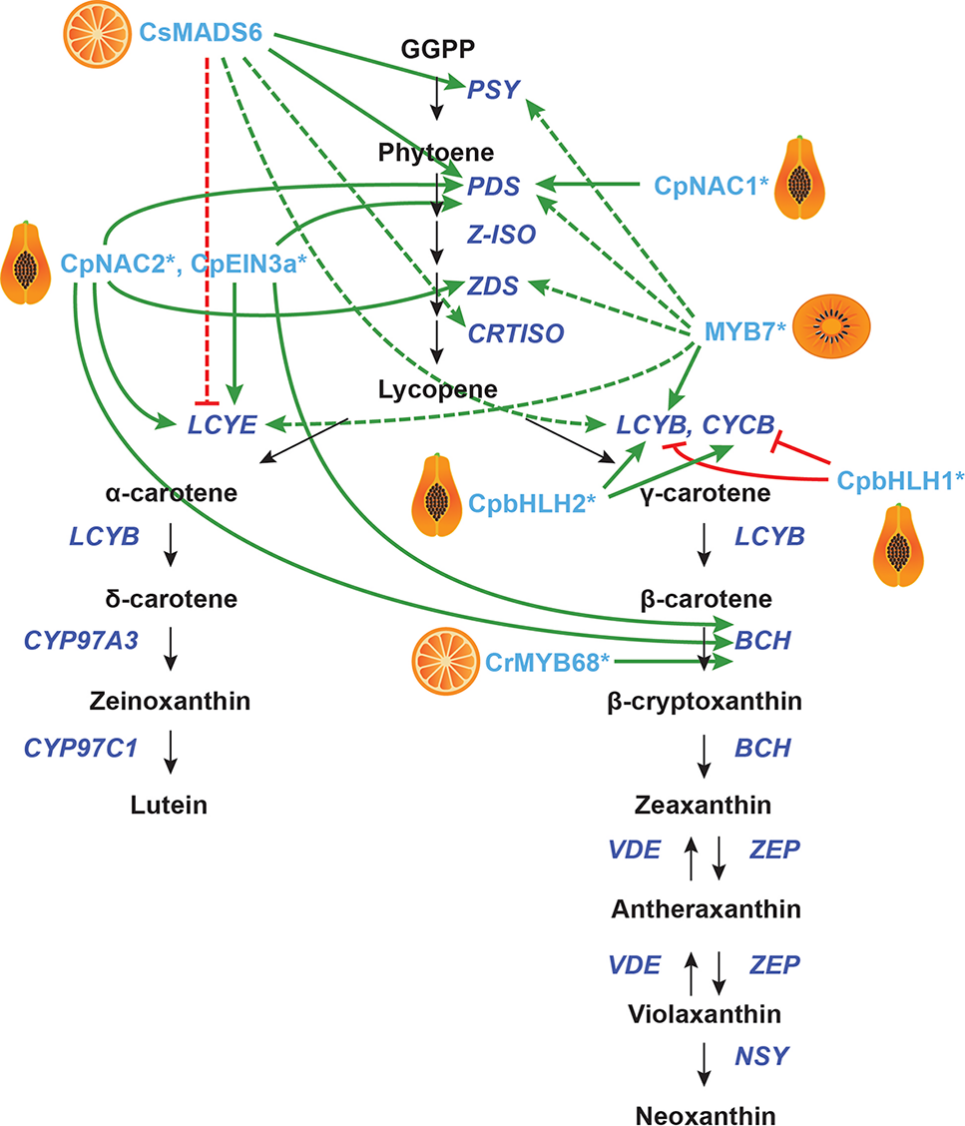
Transcriptional regulation of CBP genes in other fruits. The regulation of CBP genes in citrus, peach, papaya, and orange kiwi. The carotenoid biosynthesis pathway is shown in black, with carotenoid biosynthesis genes indicated in dark blue. Carotenoid regulators discussed in the paper are shown in light blue. Green arrows indicate positive regulation, while blunt red arrows indicate negative regulation. Solid lines show direct interactions, while dotted lines show indirect/unknown interactions.

Putative transcriptional regulators have also been identified in other climacteric fruits (**Figure 4**). In papaya (*Carica papaya*), *in vitro* and dual luciferase assays in a heterologous host (i.e., tobacco) showed that the ethylene response protein CpEIN3a binds to and activates the promoters of *CpPDS4* and *CpBCH*. Further, its interacting partner CpNAC2 binds to and activates the promoters of *CpPDS2*, *CpPDS4*, *CpZDS*, *CpLCYE*, and *CpBCH*. The interaction between CpEIN3a and CpNAC2 increases activation of these genes (Fu et al., 2017). Also in papaya, the NAC family transcription factor CpNAC1 has been shown to bind to the *CpPDS2* and *CpPDS4* promoters *in vitro* and activate them in transient assays in tobacco (Fu et al., 2016). Two other papaya transcription factors, CpbHLH1 and CpbHLH2, bind to the promoters of *CpCYCB* and *CpLCYB in vitro* and in tobacco transient assays, with CpbHLH1 acting as a repressor and CpbHLH2 as an activator (Zhou et al., 2019). However, the endogenous functions of these papaya genes are unknown.

In kiwifruit (*Actinidia deliciosa*), a promoter screen identified *AdMYB7* (among other MYBs) as a putative regulator of *AdLCYB*. The authors confirmed interaction between AdMYB7 and the *AdLCYB* promoter in a gel mobility shift assay. When *AdMYB7* was overexpressed in tobacco in a transient assay, the carotenoid content increased twofold. Stable overexpression of this gene in tobacco gave increased expression of *NbPSY*, *NbPDS*, *NbZDS*, *NbLCYB*, *NbLCYE*, and chlorophyll biosynthesis genes (Ampomah-Dwamena et al., 2019).

#### Non-Climacteric Fruits

Watermelon (*Citrullus lanatus*) fruits, like tomatoes, accumulate lycopene. However, they are non-climacteric, meaning that their ripening process is not concurrent with a spike of ethylene production and cellular respiration. The expression of watermelon homologues of *CNR*, *SlAP2a*, and *SlERF6* was correlated with ripening and carotenoid biosynthesis; however, that of *RIN*, *TAGL1*, *NAC-NOR*, *DET1*, *DDB1*, and *CUL4* was not (Grassi et al., 2013). This suggests that some regulators might be common to carotenoid-accumulating fruits, while others are potentially involved in other aspects of ripening, such as ethylene biosynthesis/perception and light sensing/plastid transition.

In citrus, a yeast one-hybrid screen using the promoters of *CsLCYB1* and *CsLYCB2* identified the MADS-box gene *CsMADS6* (a homologue of *TAGL1*), which is expressed in flowers and fruits. Overexpression of *CsMADS6* in citrus calli gave increased expression of *CsPSY*, *CsPDS*, *CsCRTISO*, *CsLCYB2*, and *CsBCH*, while transcription of *CsLCYE* was repressed. Additionally, the transcript levels of citrus *HY5* and *RAP2.2* homologues increased, while *PIF1* levels were reduced (*RIN* and *FUL* are not expressed in citrus calli). CsMADS6 can bind the promoters of *CsPSY* and *CsPDS in vitro* to activate them (Lu et al., 2018). Another citrus gene, the *R2R3-MYB* *CrMYB68*, has been shown to directly and negatively regulate *CrBCH2* by Electromobility Shift Assays and dual luciferase assays, but the endogenous function of this gene in citrus is unknown (Zhu et al., 2017b).

### Flowers

**Figure 5:**
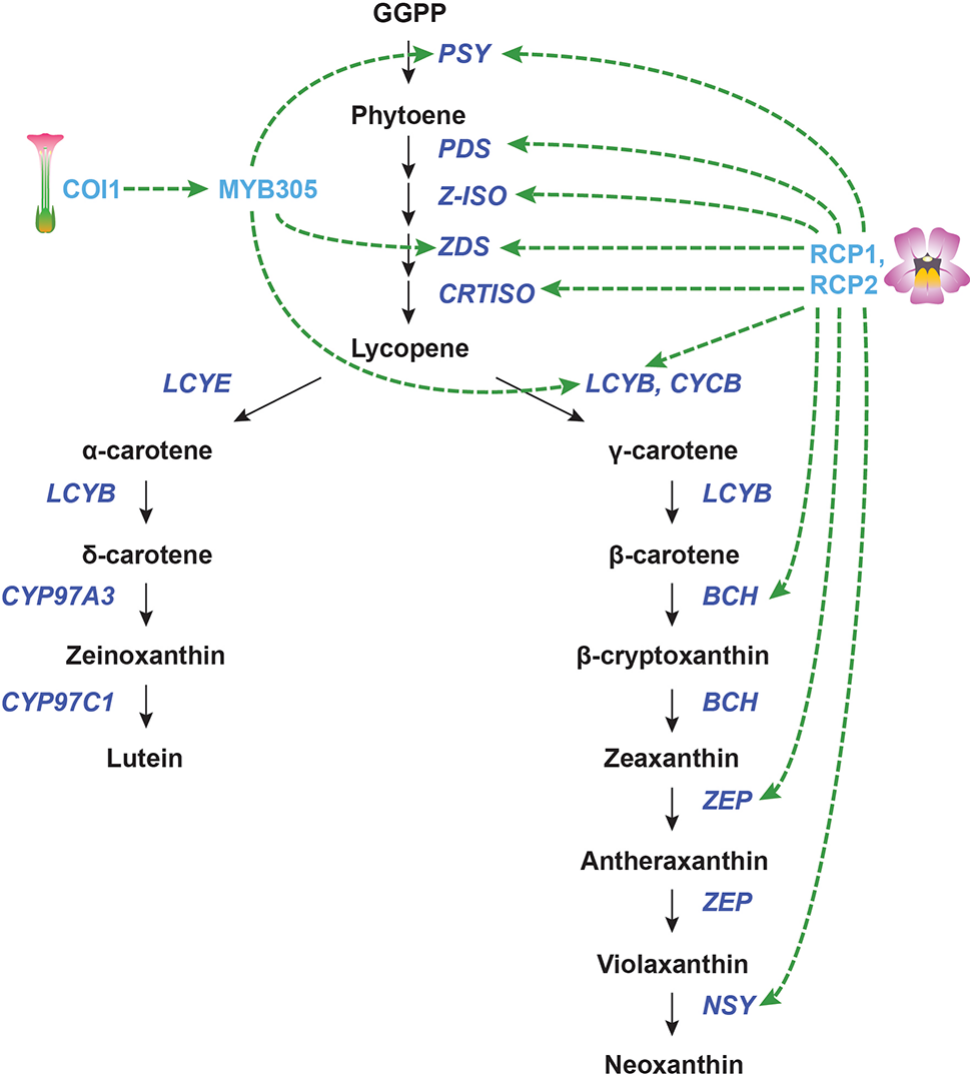
Transcriptional regulation of CBP genes in flowers. The regulation of CBP genes in *Nicotiana tabacum* and *Mimulus* species. The carotenoid biosynthesis pathway is shown in black, with carotenoid biosynthesis genes indicated in dark blue. Carotenoid regulators discussed in the paper are shown in light blue. Green arrows indicate positive regulation, while blunt red arrows indicate negative regulation. Dotted lines show indirect/unknown interactions.

The coordinated transcriptional regulation of CBP genes is largely responsible for the coloration of carotenoid-pigmented flowers (e.g., Corona et al., 1996; Moehs et al., 2001; Chiou et al., 2010; Dalal et al., 2010; Yamagishi et al., 2010; Yamamizo et al., 2010; Ohmiya, 2013; Zhang et al., 2015; Kishimoto et al., 2018; Wang and Yamagishi, 2019). However, very few genes regulating the transcription of CBP genes in flowers have been identified (**Figure 5**). Notably, none of the regulators involved in tomato fruit ripening dramatically affect flower petal color, even though tomatoes have carotenoid-pigmented flowers. This is probably because fruit and flower carotenoid biosynthesis are differentially regulated: while tomato fruits accumulate lycopene, the main carotenoid components in tomato flowers are the xanthophylls violaxanthin and neoxanthin (Galpaz et al., 2006). Most flowers studied to date primarily store xanthophylls and/or β-carotene (Ohmiya, 2011).

The F-box protein CORONATINE INSENSITIVE 1 (COI1) is necessary for the perception of jasmonic acid JA. In addition to its many other functions, COI1-mediated JA signaling has been implicated in the production of floral and extrafloral nectar. Silencing of *COI1* in *Nicotiana tabacum* not only suppresses nectar production in flowers but also decreases the amount of β-carotene in the floral nectary. The expression of *NtPSY*, *NtZDS*, and *NtLCYB* was reduced in the *COI1*-silenced lines during carotenoid accumulation (and persisted throughout development for *NtPSY*). Silencing *COI1* also strongly downregulated the *R2R3-MYB* gene *MYB305* in floral nectaries, suggesting that COI1 works upstream of this gene (Wang et al., 2014). It was previously shown that RNAi knockdown of *MYB305* causes the loss of β-carotene in floral nectaries (though gene expression of the CBP genes was not analyzed) (Liu et al., 2009). MYB305, then, may mediate the transcriptional regulation of *NtPSY*, *NtZDS*, and *NtLCY*.

In the monkeyflower species *Mimulus lewisii*, an *R2R3-MYB* gene called *Reduced Carotenoid Pigmentation 1 (RCP1)* positively regulates all of the CBP genes expressed in flowers, contributing to the bright yellow coloration of the floral nectar guides (Sagawa et al., 2016). Although this seems like a promising global regulator for carotenoid biosynthesis in flowers, the DNA binding site/s of this transcription factor is/are yet to be determined. It is unknown whether RCP1 directly or indirectly activates transcription of the CBP genes. Another gene from monkeyflowers, *RCP2*, is also necessary for carotenoid biosynthesis in petals. *RCP2* codes for a tetratricopeptide repeat protein that promotes the expression of the entire CBP, apparently through the regulation of chromoplast formation (Stanley et al., 2017). It appears that chromoplast defects in *rcp2* mutants are somehow conveyed to the nucleus through retrograde signaling, which reduces transcription of all CBP genes. Again, the mechanism for this coordinated regulation of carotenoid biosynthesis genes is still unknown, and almost certainly indirect.

### Seeds

Seed carotenoids are critical for ABA biosynthesis and seed dormancy, as well as protecting seeds from ROS damage. Therefore, carotenoids contribute to successful germination (Howitt and Pogson, 2006). In *Arabidopsis* and *Nicotiana plumbaginifolia*, it has been shown that *ZEP* transcript levels increase during seed development, peaking just before the accumulation of ABA (Audran et al., 1998; Audran et al., 2001).

**Figure 6:**
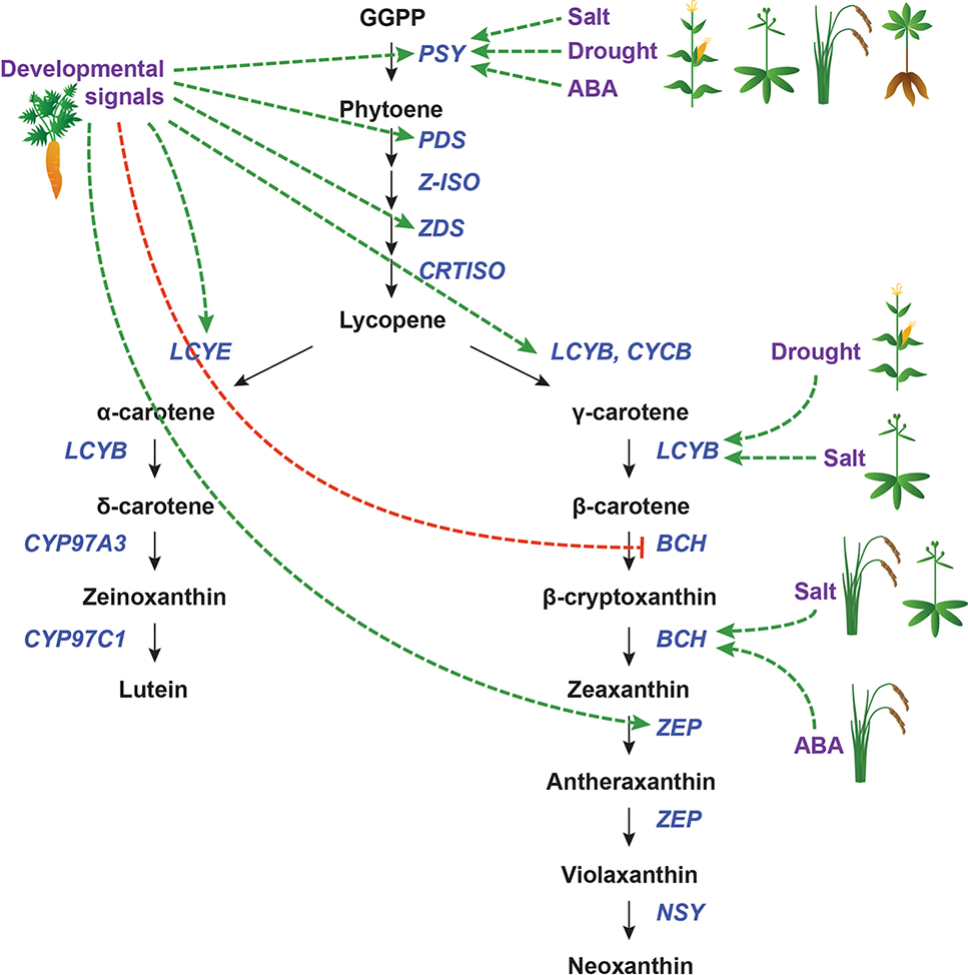
Transcriptional regulation of CBP genes in roots. The regulation of CBP genes in root tissues. Icons indicate the species (carrot, cassava, maize, rice, and *Arabidopsis*). Developmental and environmental cues are shown in purple. The carotenoid biosynthesis pathway is shown in black, with carotenoid biosynthesis genes indicated in dark blue. Carotenoid regulators discussed in the paper are shown in light blue. Green arrows indicate positive regulation, while blunt red arrows indicate negative regulation. Dotted lines show indirect/unknown interactions.

Very few transcriptional regulators of seed carotenoid biosynthesis have been identified (**Figure 6**). In maize, the endosperm P-box and AACA motif regulatory sequences are bound by P-box binding factor (PBF) and GAMYB proteins, respectively. The *ZmBCH2* promoter contains both elements and is bound *in vitro* by PBF and GAMYB (Jin et al., 2019). A transient assay in maize showed that overexpression of each transcription factor alone increased *ZmBCH2* transcript levels, but together, the effect was not additive. This regulation of *ZmBCH2* is probably tied to ABA biosynthesis, and not carotenoid accumulation, as maize seeds accumulate lutein (a separate branch of the pathway).

It is perhaps surprising that so little is known about CBP transcriptional regulation in seeds, given the developmental and economic importance of seed carotenoids. This may be because many carotenoid-containing seeds primarily accumulate lutein (e.g., wheat, maize, millet, sunflower, pumpkin, canola), and the regulation of the α-carotene branch of the pathway is little understood (Howitt and Pogson, 2006). Additionally, promoter screens for late pathway carotenoid biosynthesis genes are rarely performed and/or reported, perhaps due to a biased focus on early pathway genes like *PSY*.

### Roots

Although most roots do not produce carotenoids in appreciable amounts, the CBP is active to provide the precursors for ABA biosynthesis (Rock and Zeevaart, 1991; Bartley and Scolnik, 1995). ABA induces expression change of stress-related genes in response to dehydration (reviewed in Shinozaki and Yamaguchi-Shinozaki, 2007). Thus, the transcriptional regulation of carotenoid biosynthesis is key to water stress responses in plants. Additionally, some crop plants (e.g., sweet potatoes and carrots) accumulate large amounts of carotenoids in storage roots, where developmental signals regulate CBP genes over the course of root maturation.

#### Abiotic Stress Responses

Because roots are responsible for water and nutrient acquisition, root tissues must be able to respond to environmental cues. Of particular relevance to carotenoid biosynthesis is the sensing of and response to drought and salt stress (**Figure 6**). These mechanisms are related and overlapping (Zhu, 2002) and will thus be considered together. Rice (*Oryza sativa*) and maize each have three *PSY* paralogues, one of which (*PSY3*) appears to be strongly inducible by drought, salt, and exogenous ABA application. *OsZEP* and *ZmBCH* are also moderately upregulated by these stressors in rice and maize, respectively (Welsch et al., 2008; Li et al., 2008b).

In cassava (*Manihot esculenta*), there are also three copies of *PSY*. However, *MePSY3* is expressed at extremely low levels, and its transcription is not affected by salt or drought stress. Instead, *MePSY1* and *MePSY2*, which are normally expressed in photosynthetic tissues, are upregulated in roots to mediate the salt/drought response (Arango et al., 2010).

In *Arabidopsis*, which has only one *PSY* gene, salt stress upregulates *AtPSY* in the root but not the shoot. There is also a root-specific increase in the transcript levels of *AtBCH1*, *AtBCH2*, and *AtZEP*, but not other CBP genes. It was speculated that ABA signaling transcription factors might bind the *AtPSY* promoter preferentially in the root (Ruiz-Sola et al., 2014).

It appears that the transcriptional regulation of *PSY* is a conserved mechanism for drought/salt stress response in plants. When multiple paralogues of *PSY* are present in a genome, there may be specialization in function, perhaps mediated by differences in *cis*-regulatory elements (CREs). In cases where no specialization is evident, *PSY* regulation is tissue-specific: in both cassava and *Arabidopsis*, salt/drought stress upregulates *PSY* specifically in the root, with leaves exhibiting no change in *PSY* mRNA levels. Some downstream genes in the β-carotene branch of the CBP (which leads to ABA) are also altered by drought/salt stress, but the affected genes appear to be species-specific, with no later pathway genes being consistently responsive.

#### Storage Roots

In carrots (*Daucus carota*), the expression levels of most CBP genes (*DcPSY1/2*, *DcPDS*, *DcZDS1/2*, *DcLCYE*, *DcLCYB*, and *DcZEP*) increase over root development in several different carrot cultivars, including white carrots, which ultimately do not sequester carotenoids (Clotault et al., 2008). White carrots overexpressing a bacterial orthologue of *PSY* (c*rtB*) in roots had significantly increased carotenoid levels (though not nearly as much β-carotene as orange carrots, suggesting that other factors also mediate this process) (Maass et al., 2009). This demonstrated that transcriptional regulation could play an important role in carotenoid accumulation in carrot roots.

In Fuentes et al. (2012), carrot roots were grown either underground or in light, and the mRNA levels of multiple CBP genes were assessed. Light-grown carrots accumulated a carotenoid profile similar to that of leaves, while dark-grown carrots accumulated mostly β-carotene. The expression patterns of most CBP genes mirrored these changes, with the exception of *DcZDS1* and *DcLCYB2* (which were not affected by treatment) and *DcLCYE* (which actually increased in both treatments). As proposed before, transcriptional regulation alone cannot account for these differences. It is important to note that light induced the formation of chloroplasts instead of chromoplasts, which indicates that plastid-to-nucleus retrograde signaling may somehow regulate CBP gene expression.

### So Many Regulators, So Little Consensus

A fairly large number of putative transcriptional regulators of carotenoid biosynthesis have been identified from various species and tissue types (**Table S1**), mostly in the past decade. A conundrum emerges from this otherwise exciting progress: why so many candidate regulators but so little consensus? Almost every tissue type in every species studied to date seems to utilize a different group of transcriptional regulators for carotenoid biosynthesis. Perhaps it is not too surprising that the CBP is differentially regulated in various tissue types, as carotenoids serve very different functions in different organs (e.g., in leaves as essential components of the photosynthetic apparatus vs. in flowers and fruits as secondary metabolites), but it is puzzling that each species seems to have evolved its own carotenoid regulators. In our opinion, this conundrum exists at least in part because of the following challenges:

1. The endogenous functions of some of these putative transcriptional regulators have not been verified through knockout or knockdown experiments (e.g., *CpEIN3a*, *CpNAC1/2*, *CrMYB68*, *CsMADS6*, *CpbHLH1/2*, *AdMYB7*, *ZmPBF*, *ZmGAMYB*). These regulators were usually identified through transcriptome-based co-expression analyses or yeast one-hybrid screens using promoters of CBP genes. Interactions between these regulators and their DNA binding sites in the CBP gene promoters were often tested by *in vitro* gel shift assays and/or dual luciferase assays. Sometimes these regulators were further characterized by transient or stable overexpression in a heterologous host (e.g., tobacco). However, one should be cautious when interpreting these results, as heterologous expression can sometimes be uninformative or even misleading (Kramer, 2015). Before knockout or knockdown data become available, we think these genes should be regarded as “candidate” instead of *bona fide* carotenoid regulators.
2. Most putative regulators were identified from ripening fruits, especially tomato (e.g., *TAGL1*, *RIN*, *FUL1*, *FUL2*, *SlMADS1*, *SlNAC4*, *SlAP2a*, *SGR1*, *SlHDA3*), making it very difficult to disentangle these regulators’ influence on carotenoid biosynthesis from their other ripening roles, which are largely mediated through ethylene signaling. In fact, most of the putative carotenoid regulators identified in tomato are components of the ethylene signaling network (Li et al., 2019). Therefore, it would not be unexpected if these tomato fruit ripening genes do not regulate carotenoid biosynthesis in other tissue types or in non-climacteric fruits.
3. Current major model systems for carotenoid regulation are somewhat unusual or at least not representative. For example, the best genetic model system, *Arabidopsis*, does not produce chromoplast-containing tissues. The foremost fruit model, tomato, accumulates lycopene and is climacteric, whereas fruits of many other plant species (e.g., orange, papaya) accumulate abundant downstream products (e.g., β-carotene and xanthophylls). These limitations raise the question whether the knowledge gained from these systems is widely applicable to other plant species.
4. Minimal effort has been put into testing whether the function of a certain regulator identified from one species is conserved in another species. So far, only the PIF1/HY5 regulatory module has been shown to play a role in carotenoid biosynthesis during both *Arabidopsis* photomorphogenesis and the onset of tomato fruit ripening. Even in this case, it is unclear whether PIF1/HY5 function at the onset of tomato fruit ripening is an ancestral feature of all fleshy fruits or was accidentally co-opted from the photomorphogenesis network just in tomatoes.

In addition to these challenges, there are also many gaps in our understanding of transcriptional regulation of carotenoid biosynthesis. For example, we know very little about what regulates most CBP genes downstream of *PSY* in photosynthetic tissues, even in *Arabidopsis*; we know virtually nothing about the transcriptional regulators of CBP genes in the roots of any model systems; we know only three regulators in flowers (i.e., COI1/MYB305, RCP1, and RCP2), and even for these, we know nothing about their functional mechanisms; we know a variety of phytohormones affecting CBP gene expression, but we do not know which transcriptional regulators relay their signals to CBP genes.

## Future Perspectives

The challenges and knowledge gaps discussed above present wonderful opportunities for future carotenoid research. We think the following research directions will be fruitful in understanding how carotenoid biosynthesis is controlled at the transcriptional level:

1. Testing the function of known putative carotenoid regulators (**Table S1**) across multiple species with well-developed genetic/genomic resources and functional tools. For example, generating knockdown transgenic lines or Clustered Regularly Interspaced Short Palindromic Repeats (CRISPR) mutants of the *SDG8* orthologue in readily transformable species like tomatoes or monkeyflowers would be a straightforward way to test whether the role of this gene in *CRTISO* regulation is conserved across angiosperms or just an oddity in *Arabidopsis*. Likewise, it would be interesting to generate knockdown/knockout lines of *RCP1* or *RCP2* in tomatoes to see whether tomato flower color changes.
2. Identifying regulators of late pathway CBP genes using promoter screens. The recent study in maize (Jin et al., 2019) where two new regulators were identified using the *ZmBCH2* promoter is a good example. Besides traditional yeast one-hybrid screens, recently developed methods such as CAPTURE (CRISPR Affinity Purification *in siTU* of Regulatory Elements) can be used to identify both transcription factors and chromatin remodelers at a particular promoter site with high specificity (Liu et al., 2017).
3. Discovering key CREs of the CBP genes in various species. Databases such as PLACE (Higo et al., 1999) and PlantCare (Lescot et al., 2002), in conjunction with phylogenetic shadowing methods (Blanchette and Tompa, 2002), are extremely useful in predicting CREs *in silico*. Additionally, for species that are transformable, a powerful new way to discover CREs *in vivo* is CRISPR/Cas9 genome editing with multiple guide RNAs targeted to a gene promoter (e.g., eight guide RNAs in Rodríguez-Leal et al., 2017). This method can generate a wide range of mutant alleles with deletions of various lengths within the promoter region; and because this method does not rely on *a priori* knowledge of sequence motifs, it allows the discovery of novel CREs.
4. Integrating multi-omics data (genomics, transcriptomics, proteomics, metabolomics, etc.) towards a more comprehensive understanding of CBP gene expression. With the rapid advances in generating large quantities of high-quality data as well as sophisticated bioinformatics methods and analytical tools, the systems biology approach will allow us to uncover correlations between metabolome and transcriptome profiles, to identify candidate transcriptional regulators of biosynthetic genes in co-expression modules, and to map regulatory network interactions (e.g., Amiour et al., 2012; Maruyama et al., 2014; Balazadeh et al., 2014; Larsen et al., 2016). We envision that this integrative approach will be particularly helpful in elucidating the “missing” regulators that relay various hormone cues to CBP genes. There are many “omics” resources and databases that could be used for these purposes (Mochida and Shinozaki, 2011; Rai et al., 2017), but there are also many challenges to integrating such data. Experimental design and data quality/curation must be taken into account when combining multiple omics resources (Cavill et al., 2016; Helmy et al., 2016).
5. Broadening the diversity of “model” systems. For example, citrus would be an excellent system to complement the existing tomato fruit model because it is non-climacteric and accumulates various carotenoids beyond lycopene. In addition, carotenoid-containing, embryogenic citrus calli can be readily produced and transformed (Lu et al., 2018), making them a powerful tool for rigorous tests of gene function. As high-quality genome assemblies and genome editing technologies become more and more accessible, it is not difficult to envision the development of even brand-new model species with interesting/economically important carotenoid phenotypes in the near future.

We believe that these research avenues will lead to many more exciting discoveries in the coming years, which will not only contribute new knowledge on the transcriptional regulation of carotenoid biosynthesis but also likely have a significant impact on carotenoid biofortification of crop plants. So far, most of the efforts towards enhancing carotenoid biosynthesis or engineering novel carotenoid products in staple crops have focused on CBP genes (e.g., aSTARice; Zhu et al., 2018). However, effective biofortification often requires transferring multiple CBP genes simultaneously to the host plant and expressing these transgenes in a coordinated fashion, the latter being a particular challenge in metabolic engineering (Nielsen and Keasling, 2016). Building both transcriptional regulators and CBP genes into a synthetic biology framework will allow us to better coordinate the expression of multiple CBP genes, to make quantitative predictions of metabolic flux, and to rationally design optimal genetic circuits with maximal phenotypic outputs.

## Author Contributions

LS and Y-WY wrote the manuscript.

## Funding

Our work on carotenoids is supported by the National Science Foundation (IOS-1558083, IOS-1827645).

## Conflict of Interest Statement

The authors declare that the research was conducted in the absence of any commercial or financial relationships that could be construed as a potential conflict of interest.
