# Supplementary material for: Transcriptional Regulation of Carotenoid Biosynthesis in Plants: So Many Regulators, So Little Consensus
Source: Front Plant Sci. 2019 Aug 9;10:1017. doi: 10.3389/fpls.2019.01017 (PMC6695471; doi:10.3389/fpls.2019.01017)
Supplement: Supplementary file 1 [file Table_1.docx]

**Table S1. Transcriptional regulators of carotenoid biosynthesis.** A summary of regulators discussed in this review, giving protein name, class, system, and carotenoid biosynthesis pathway (CBP) genes positively/negatively regulated. Information about regulatory mechanisms is provided in the D/I/U, T/V, E/H, and Interacting factors columns. References for each regulator are provided. Regulators whose mechanisms are direct and have been demonstrated *in vivo* in their endogenous systems are bolded. Note: if a regulator affects some CBP genes directly and others indirectly/unknown, indirect/unknown regulation is shown in parentheses.

| **Regulator** | **Class** | **Endogenous**  **system** | **CBP genes**  **positively regulated** | **CBP genes negatively regulated** | **D/I/U*** | **T/V^†^** | **E/H^‡^** | **Interacting factors** | **Reference** |
| --- | --- | --- | --- | --- | --- | --- | --- | --- | --- |
|  |  |  |  |  |  |  |  |  |  |
| **PIF1** | bHLH | *Arabidopsis* | *-* | *PSY* | D | V | E | DET1, DDB1, CUL4, PAR1, DELLAs | Toledo-Ortiz et al., 2010; Llorente et al., 2017; Rodriguez-Villalon et al., 2009; Cheminant et al., 2011 |
| **SlPIF1a** | bHLH | Tomato | *-* | *PSY1* | D | V | E | - | Llorente et al., 2015 |
| **HY5** | bZIP | *Arabidopsis* | *PSY* | *-* | D | V | E | COP1, DDB1, CUL4, DELLAs | Toledo-Ortiz et al., 2014; Llorente et al., 2017; Rodriguez-Villalon et al., 2009; Cheminant et al., 2011 |
| DRL1 | NAC | *Vitis* | *-* | *ZEP* | U | - | - | - | Zhu et al., 2019 |
| RAP2.2 | AP2/ERF | *Arabidopsis* | *PSY, PDS* | *-* | D | T | - | - | Welsch et al., 2007 |
| SlAP2a | AP2/ERF | Tomato | *PSY1, CRTISO, BCH, PDS1* | *ZEP1, CYCB* | U | - | - | SlCMB1 | Chung et al., 2010, Karlova et al., 2011 |
| **SDG8** | HMT | *Arabidopsis* | *CRTISO, LCYE* | *-* | D | V | E | - | Cazzonelli et al., 2009 |
| TAGL1 | MADS-box | Tomato | *PSY1* | *LCYB, CYCB* | U | - | - | RIN, FUL2, SlCMB1 | Vrebalov et al., 2009; Fujisawa et al., 2014 |
| CsMADS6 | MADS-box | *Citrus* | *PSY, PDS (CRTISO, LCYB2, BCH)* | *(LCYE)* | D+(I) | V | H | - | Lu et al., 2018 |
| **RIN** | MADS-box | Tomato | *PSY1, Z-ISO, CRTISO (PSY2, ZDS)* | *LCYE, LCYB, ZEP* | D+(I) | V | E | FUL1, FUL2, TAGL1, SlCMB1, SlMADS1, SlFYFL | Fujisawa et al., 2011, 2012, 2013, 2014; Martel et al., 2011; Zhong et al., 2013 |
| **FUL1** | MADS-box | Tomato | *PSY1, Z-ISO, CRTISO, BCH* | *LCYB, ZEP* | D | V | E | RIN | Shima et al., 2013; Fujisawa et al., 2014 |
| **FUL2** | MADS-box | Tomato | *Z-ISO, CRTISO, BCH* | *LCYB, ZEP* | D | V | E | TAGL1, RIN | Fujisawa et al., 2014 |
| SlMADS1 | MADS-box | Tomato | *-* | *PSY1* | U | - | - | RIN, SlFYFL, SlCMB1 | Dong et al., 2013 |
| SlFYFL | MADS-box | Tomato | *-* | *PSY1, PDS, ZDS* | U | - | - | RIN, SlMADS1, SlJOINTLESS | Xie et al., 2014 |
| SlCMB1 | MADS-box | Tomato | *PSY1, PDS* | *CYCB, LCYE, LCYB* | U | - | - | RIN, SlMADS1, TAGL1, AP2a | Zhang et al., 2018 |
| TERF1 | AP2/ERF | Tomato | *ZDS* | *-* | U | - | - | - | Wu et al., 2019 |
| SlPti4 | AP2/ERF | Tomato | *-* | *CYCB* | U | - | - | - | Sun et al., 2018 |
| SlBZR1 | BR response TF | Tomato | *PSY1, ZDS* | *-* | U | - | - | - | Liu et al., 2014 |
| SlNAC4 | NAC | Tomato | *PSY1* | *CYCB, LCYE, LCYB* | U | - | - | RIN, NOR | Zhu et al., 2013 |
| SlNAC1 | NAC | Tomato | *(CYCB, LCYE, LCYB)* | *PSY1* | D+(U) | T | - | - | Ma et al., 2014; Meng et al., 2016 |
| SlARF2 | ARF | Tomato | *PSY1, PDS, ZDS* | *LCYB1, LCYB1, CYCB* | U | - | - | - | Hao et al., 2015 |
| SlPRE2 | bHLH (atypical) | Tomato | *-* | *PSY1, PDS, ZDS* | U | - | - | - | Zhu et al., 2017 |
| SlBBX20 | B-box | Tomato | *PSY1, VDE* | *-* | D | T | - | DET1 | Xiong et al., 2019 |
| ClpR1 | Clp protease | Tomato | *LCYB, CYCB, CYP97C11* | *-* | U | - | - | - | D’Andrea et al., 2018 |
| SGR1 | STAY-GREEN | Tomato | *-* | *PSY1* | U | - | - | PSY1 | Luo et al., 2013 |
| SlIPT4 | IPT | Tomato | *Z-ISO, ZDS* | *PSY1, PDS, CRTISO, LCYB, LCYE* | U | - | - | - | Zhang et al., 2018 |
| CNR | SPL | Tomato | *PSY1* | *-* | U | - | - | - | Zhong et al., 2013 |
| SlHDA3 | HDA | Tomato | *CYCB, LCYB, LCYE* | *PSY1* | U | - | - | - | Guo et al., 2018 |
| CpEIN3a | EIN3/EIL | Papaya | *PDS4, BCH* | *-* | D | V | H | CpNAC2 | Fu et al., 2017 |
| CpNAC2 | NAC | Papaya | *PDS2, PDS4, ZDS, LCYE, BCH* | *-* | D | V | H | CpEIN3a | Fu et al., 2017 |
| CpNAC1 | NAC | Papaya | *PDS2, PDS4* | *-* | D | V | H | - | Fu et al., 2016 |
| CpbHLH1 | bHLH | Papaya | *-* | *CYCB, LCYB* | D | V | H | - | Zhou et al., 2019 |
| CpbHLH2 | bHLH | Papaya | *CYCB, LCYB* | *-* | D | V | H | - | Zhou et al., 2019 |
| AdMYB7 | MYB | Kiwifruit | *LCYB (PSY, PDS, ZDS, LCYE)* | *-* | D+(U) | T | - | - | Ampomah-Dwamena et al., 2018 |
| CrMYB68 | MYB | *Citrus* | *BCH2* | *-* | D | V | H | - | Zhu et al., 2017 |
| COI1 | F-box | *Nicotiana* | *PSY, ZDS, LCY* | *-* | U | - | - | - | Wang et al., 2014 |
| RCP1 | MYB | *Mimulus* | *PSY1, PDS1, PDS2, Z-ISO, ZDS1, ZDS2, CRTISO, LCYB1, BCH1, ZEP1, ZEP2, NSY1* | *-* | U | - | - | - | Sagawa et al., 2016 |
| RCP2 | TPR | *Mimulus* | *PSY1, PDS1, PDS2, Z-ISO, ZDS1, ZDS2, CRTISO, LCYB1, BCH1, ZEP1, ZEP2, NSY1* | *-* | U | - | - | - | Stanley et al., 2017 |
| PBF | P-box | Maize | *BCH2* | *-* | D | T | - | - | Jin et al., 2018 |
| GAMYB | MYB | Maize | *BCH2* | *-* | D | T | - | - | Jin et al., 2018 |

*D/I/U refers to the regulatory mechanism (Direct/Indirect/Unknown)

**^†^**T/V indicates whether the regulatory mechanism has been shown to function *in vitro* (T) or *in vivo* (V)

**^‡^**E/H designates whether the regulatory mechanism has been shown to function in an endogenous (E) or heterologous (H) system

Note: LeHB-1 and SlZFP2 from the main text are not included in this table because their effects on the transcription of CBP genes have not been reported.
